# Survival of uncemented acetabular monoblock cups: Evaluation of 210 hips in the Swedish Hip Arthroplasty Register

**DOI:** 10.3109/17453674.2012.688726

**Published:** 2012-06-04

**Authors:** Rüdiger J Weiss, Nils P Hailer, André Stark, Johan Kärrholm

**Affiliations:** ^1^Department of Molecular Medicine and Surgery, Section of Orthopaedics and Sports Medicine, Karolinska Institutet, Karolinska University Hospital, Stockholm; ^2^Department of Orthopaedics, Institute of Surgical Sciences, Uppsala University Hospital, Uppsala; ^3^Departement of Clinical Sciences, Karolinska Institutet, Danderyd Hospital, Stockholm; ^4^Department of Orthopaedics, Institute of Clinical Sciences at Sahlgrenska Academy, Gothenburg University, Gothenburg, Sweden; Correspondence: rudiger.weiss@karolinska.se

## Abstract

**Background and purpose:**

Monoblock acetabular cups represent a subtype of uncemented cups with the polyethylene liner molded into a metal shell, thus eliminating—or at least minimizing—potential backside wear. We hypothesized that the use of monoâ€‹block cups could reduce the incidence of osteolysis and aseptic loosening, and thus improve survival compared to modular designs.

**Patients and methods:**

We identified all 210 primary total hip arthroplasty (THA) procedures in the Swedish Hip Arthroplasty Register that used uncemented monoblock cups during the period 1999–2010. Kaplan-Meier and Cox regression analyses with adjustment for age, sex, and other variables were used to calculate survival rates and adjusted hazard ratios (HRs) of the revision risk for any reason. 1,130 modular cups, inserted during the same time period, were used as a control group.

**Results:**

There was a nearly equal sex distribution in both groups. Median age at the index operation was 47 years in the monoblock group and 56 years in the control group (p < 0.001). The cumulative 5-year survival with any revision as the endpoint was 95% (95% CI: 91–98) for monoblock cups and 97% (CI: 96–98) for modular cups (p = 0.6). The adjusted HR for revision of monoblock cups compared to modular cups was 2 (CI: 0.8–6; p = 0.1). The use of 28-mm prosthesis heads rather than 22-mm heads reduced the risk of cup revision (HR = 0.2, CI: 0.1–0.5; p = 0.001).

**Interpretation:**

Both cups showed good medium-term survival rates. There was no statistically significant difference in revision risk between the cup designs. Further review of the current patient population is warranted to determine the long-term durability and risk of revision of monoblock cup designs.

The use of uncemented components in total hip arthroplasty (THA) continues to increase in many countries that provide population-based registry data (AOA 2009, Swedish Hip Arthroplasty Register 2009, National Joint Registry of England and Wales 2010). Many of these registers report that survival of uncemented components is at best equal to that of cemented THA, but inferior survival rates of uncemented THA have been reported (Hailer et al. 2010). The inferior survival of uncemented THA has mainly been attributed to revision due to aseptic cup loosening or periprosthetic femoral fracture.

If cup failure is not evident on radiographs, the decision to perform a cup revision in the presence of wear and osteolysis or during a stem revision is complex and includes the surgeon's judgement of the future development of liner wear and osteolysis. Liner wear depends on the type of polyethylene used for its production, and on the processing of the polyethylene resin, including melting and irradiation procedures. Other parameters such as head diameter and material also influence wear. Last but not least, fixation of the liner to its metal shell influences backside wear, which has been recognised as an important contributor to liner wear (Sculco 2002, Young et al. 2002).

There are numerous examples of inferior locking mechanisms that permit micromotions of the liner relative to the shell, which in time will lead to liner rotation and sometimes even dislocation (Blacha 2004). In addition, the debris generated at the backside of the liner has direct access to subchondral bone through screw and dome holes. This direct access may contribute to an increased prevalence of acetabular osteolysis around modular cups with holes (Huk et al. 1994).

Monoblock cups are a distinct subtype of uncemented cups that are defined as acetabular components where the polyethylene liner has been molded into a metal shell prior to surgery, thus eliminating or at least minimizing potential backside wear. Early monoblock designs consisted of polyethylene molded into a titanium fiber mesh (Morscher and Masar 1988) or a solid titanium shell. More recent designs have made use of polyethylene liners in a porous tantalum metal shell (Meneghini et al. 2010). The putative advantages of monoblock designs have to be weighed against drawbacks such as the inability to assess proper seating of a component into its bony acetabular bed due to the absence of central screw holes that are uniformly present in modular cup designs. A further disadvantage may be that elevated liners cannot be used to prevent dislocation. Finally, liner wear in modular designs can be dealt with by liner exchange alone, which is not possible in monoblock cups.

We investigated revision rates of monoblock cups used in primary THA that were registered in the Swedish Hip Arthroplasty Register, using a well-documented modular uncemented acetabular cup as a reference. We hypothesized that elimination of backside wear in monoblock cups would reduce the incidence of osteolysis and aseptic loosening, thus improving survival of such designs compared to modular uncemented designs.

## Patients and methods

### Source of data

Data were extracted from the Swedish Hip Arthroplasty Register, which collects patient-based information on hip replacement surgery on a nationwide basis in Sweden. Every Swedish citizen has a unique personal identification (social security) number. This number is linked to information on all changes important for follow-up, such as date of emigration or date of death. All reoperations (any secondary operation of the hip) and revisions (exchange or removal of any of the components) are continuously reported by all operating units in Sweden. The Register covers 98–99% of all primary hip replacement surgical procedures in Sweden, whereas the coverage of revision hip arthroplasties has been estimated to be 94% (Söderman et al. 2000).

### Study population

We identified all primary THAs using uncemented monoblock acetabular components that were registered in the Swedish Hip Arthroplasty Register between 1999 and 2010 (n = 210 hips). Two cup designs had been used, the Morscher press-fit acetabular cup (Sulzer Orthopaedics Ltd., Baar, Switzerland; n = 129 hips) (Morscher and Masar 1988, Morscher et al. 1997) and the Trabecular Metal Monoblock acetabular cup system (Zimmer Inc., Warsaw, IN; n = 81 hips). As a reference group, we extracted data on the modular Trilogy cup (Zimmer Inc.; n = 1,130 hips) from the Register. This hemispherical press-fit shell was chosen because it represents one of the most commonly used uncemented cup designs in Sweden. The Trilogy cup is made of titanium alloy, available with or without hydroxyapatite (HA) coating. Only cups without HA were included in the reference group since (1) the monoblock cup designs investigated had no such coating, and (2) a previous study has suggested that HA coating may be associated with increased risk of revision (Lazarinis et al. 2010). The monoblock and modular cups were combined with either cemented or uncemented stems, resulting in totally uncemented or hybrid systems (Table 1).

**Table 1.** Baseline characteristics

	Study group	Control group
	(n = 210)	(n = 1,130)
Cup design		
Morscher press-fit cup	129 (61%)	–
Trabecular Metal Monoblock cup	81 (39%)	–
Trilogy cup	–	1,130 (100%)
Median age at surgery, years	47 (17–83)	56 (20–90)
Sex		
Male	101 (48%)	577 (51%)
Female	109 (52%)	553 (49%)
Primary diagnosis		
Primary osteoarthritis	106 (51%)	839 (74%)
Inflammatory disease	21 (10%)	34 (3%)
Pediatric hip disease	63 (30%)	154 (14%)
Idiopathic femoral head necrosis	15 (7%)	53 (5%)
Other	5 (2%)	50 (4%)
Surgical approach		
Posterior	188 (90%)	252 (22%)
Anterior	19 (9%)	791 (70%)
Missing	3 (1%)	87 (8%)
Shell holes		
Non-holed	–	2 (0%)
Multi-holed	–	59 (5%)
Cluster-holed	–	1,069 (95%)
Highly crosslinked polyethylene		
No	210 (100%)	539 (48%)
Yes	–	573 (51%)
Missing	–	18 (1%)
Head size		
22 mm	2 (1%)	65 (6%)
28 mm	159 (76%)	1,036 (92%)
≥ 32 mm	49 (23%)	11 (1%)
Missing	–	18 (1%)
Type of stem fixation		
Uncemented	202 (96%)	967 (86%)
Cemented	7 (3%)	161 (14%)
Missing	1 (1%)	2 (0%)

### Statistics

Descriptive statistics used median values (with range). Follow-up started on the day of primary THA and ended on the day of revision, death, emigration, or on December 31, 2010, whichever came first. Kaplan-Meier survival analysis was performed with the type of cup as the independent factor and revision for any reason as the endpoint. The log-rank test (Mantel-Cox) was used to investigate whether the study and control groups differed significantly from each other. Moreover, a Cox proportional hazards model was used to analyze the risk of revision for any reason. The results were expressed as hazard ratios (HRs) with 95% confidence intervals (CIs). With a simple Cox regression model (unadjusted), we analyzed the following variables: cup design (monoblock or modular), age (< 50, 50–59, 60–75, > 75 years), sex, primary diagnosis before arthroplasty (primary osteoarthritis (OA), inflammatory disease (e.g. rheumatoid arthritis, M. Bechterew), pediatric hip disease, idiopathic femoral head necrosis, and other diagnoses), type of stem fixation (cemented or uncemented), highly crosslinked liner polyethylene (yes or no), surgical approach, and prosthesis head size. Later, all variables were mutually adjusted for in a multiple Cox regression model. The assumption of proportional hazards was investigated by graphs of the log minus log survivor function against log t over grouped values of the covariates. The Cox regression model was fitted with restricted follow-up and indicated that there was no departure from the proportional hazards assumption during the first 9 years of follow-up. After 6 years, the number of cases in the monoblock cohort was less than 50. Thus, follow-up was restricted by censoring implants still at risk beyond 6 years (Ranstam et al. 2011). In patients with bilateral THAs, both sides were included in the analysis, as other studies have shown that this has no significant effect on the risk of failure (Lie et al. 2004, Hailer et al. 2010). Differences between numerical data were analyzed using the Mann-Whitney U-test and differences between categorical data were analyzed using the Chi-squared test. The level of significance was set at p ≤ 0.05. All statistical analyses were performed using the PASW statistics package version 18.

## Results

### Characteristics of the study population

There was an almost equal sex distribution in both groups. Median age at the index operation was 47 (17–83) years in the patients with monoblock cups as compared to 56 (20–90) years in the controls (p < 0.001). Primary osteoarthritis was diagnosed in more than half of the patients in both groups, but the diagnosis of primary osteoarthritis was more common in the control group (p < 0.001). In contrast, the diagnosis of previous pediatric hip disease was more common in the monoblock group (p < 0.001). 90% of the patients with monoblock cups were operated using a posterior approach and 70% of the controls were operated with an anterior approach (p < 0.001). Highly crosslinked polyethylene liners were used in half of the cases in the reference group, but were not available for patients with monoblock cups. Most patients in both the study and the reference group were operated with 28-mm prosthesis heads and uncemented stems (Table 1). The median follow-up time was 4 (0–12) years for the monoblock cups and 6 (0–12) years for the modular cup.

### Revision risk—monoblock vs. modular cup

The cumulative 5-year survival with any revision as the endpoint was 95% (CI: 91–98) for monoblock cups and 97% (96–98) for the modular cups (Figure). Kaplan-Meier survival analysis with the log-rank test did not show any statistically significant difference between the study group and the control group (p = 0.6). The crude hazard ratio of monoblock cups for cup revision for any reason without adjustment for covariates was 1.3 (CI: 0.6–2.7). Subsequently, hazard ratios of each covariate mentioned above were calculated. In this analysis, other diagnoses compared with primary osteoarthritis were associated with an increased risk of revision (HR = 5, CI: 2–12). The use of 28-mm prosthesis heads as compared to 22-mm heads reduced the risk of cup revision (HR = 0.3, CI: 0.1–0.6) (Table 2).

**Figure:**
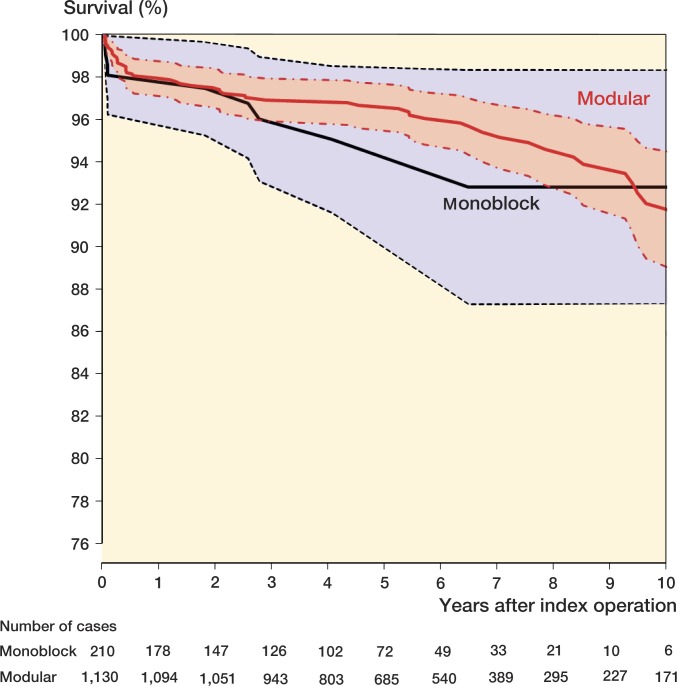
Kaplan-Meier analysis (with 95% CI) of monoblock and modular cups with revision for any reason as the endpoint.

**Table 2.** Cox regression analysis, hazard ratios (HRs) of cup revision for any reason

			Simple Cox regression **^a^**			Multiple Cox regression **^b^**		
Covariate	No. of cases	No. of revisions	HR	95% CI	p-value	HR	95% CI	p-value
Cup								
Monoblock	210	8	1.3	0.6–2.7	0.6	2.2	0.8–5.8	0.1
Modular	1,130	40	Ref.			Ref.		
Age								
< 50	415	16	Ref.			Ref.		
50–59	517	15	0.7	0.4–1.5	0.4	0.9	0.4–1.9	0.7
60–75	381	15	1.1	0.5–2.2	0.8	1.0	0.4–2.4	0.9
> 75	27	2	2.2	0.5–9.7	0.3	1.6	0.3–8.0	0.6
Sex								
Male	678	24	1.0	0.6–1.7	0.9	1.5	0.8–2.7	0.2
Female	662	24	Ref.			Ref.		
Primary diagnosis								
Primary osteoarthritis	945	26	Ref.			Ref.		
Inflammatory disease	55	2	1.3	0.3–5.6	0.7	1.1	0.2–4.9	0.9
Pediatric hip disease	217	9	1.5	0.7–3.1	0.3	0.9	0.3–2.2	0.7
Idiopathic femoral head necrosis	68	4	2.1	0.7–6.1	0.2	2.1	0.7–6.1	0.2
Other	55	7	5.3	2.3–12	0.001	4.4	1.8–11	0.001
Surgical approach								
Posterior	440	16	1.0	0.5–1.8	1.0	1.1	0.5–2.4	0.9
Anterior	810	29	Ref.			Ref.		
Highly crosslinked polyethylene								
Yes	573	22	1.3	0.7–2.3	0.4	1.4	0.7–3.0	0.4
No	749	26	Ref.			Ref.		
Head size								
22 mm	67	8	Ref.			Ref.		
28 mm	1,195	40	0.3	0.1–0.6	0.001	0.2	0.1–0.5	0.001
> 32 mm	60	–	0	0–9.7	1.0	0	0–1.7	1.0
Type of stem fixation								
Uncemented	1,169	42	1.4	0.6–3.5	0.5	1.1	0.4–3.1	0.9
Cemented	168	5	Ref.			Ref.		

**^a^** crude HR;

**^b^** adjusted HR (all covariates mentioned above were entered in the Cox analysis).

In a second step, we calculated the adjusted risk of cup revision (multiple Cox regression analysis) and found no major changes compared to the crude hazard ratios given above. There was still no statistically significant difference in revision risk comparing monoblock cups with the modular cups (HR = 2, CI: 0.8–6) (Table 2).

## Discussion

We found good medium-term survival rates for both cup designs and no statistically significant difference in the risk of revision. The concept of monoblock cups is attractive, with encouraging results published in the literature and theoretical advantages over modular acetabular components. Excellent 10-year results were described after the use of the non-modular porous-coated Morscher cup. Of 335 THAs performed with this cup, none had required cup revision due to aseptic loosening after a mean follow-up of 10 years, and with cup revision for any reason, the 10-year survival rate was 99% (Garavaglia et al. 2011). In that study, no radiolucencies were seen around the cups, whereas osteolytic defects were found around 8% of the stems investigated. Similarly encouraging results were described after a minimum of 9 years follow-up of 125 THAs using the Morscher cup, where none of the cups were revised because of aseptic loosening or osteolysis, and 3 were revised for other reasons (Gwynne-Jones et al. 2009). Berli et al. (2007) reported the 15-year results of 280 hips implanted with the Morscher cup, quoting a survival of 98% for aseptic loosening and 95% overall. A different monoblock implant, the titanium-coated RM acetabular component, showed 94% survival after 20 years with cup revision due to aseptic loosening as endpoint (Ihle et al. 2008). A series of 127 THAs using a hydroxyapatite-coated version of the RM cup had a 98% 10-year survival with cup revision for any reason as the endpoint (Ali and Kumar 2003).

A more recent development, a porous tantalum monoblock cup, was followed in 151 hips for a minimum of 8 years, and no cup revision occurred during this period; there was also no evidence of osteolytic lesions (Macheras et al. 2009). Malizos et al. (2008) followed 223 consecutive patients who were operated with the TMT acetabular component, and documented a survival rate of 99% at a mean follow-up time of 5 years. This type of monoblock design has a lower stiffness than a cup with a solid metal backing, and therefore allows a more physiological and uniform load transfer to the surrounding bone (Bobyn et. al 2004).

A randomized comparison of a porous tantalum monoblock cup with a conventional uncemented modular cup by radiostereometry showed that both implants provided excellent primary stability, and that the monoblock implant had slightly lower rotation along the transverse axis (Baad-Hansen et al. 2011). 51 patients operated with the same cup showed no evidence of retroacetabular osteolysis when investigated by computed tomography after 10 years (Moen et al. 2011). After a mean follow-up of 5 years, Young et al. (2002) reported reduced wear and a rate of osteolysis of 2% in monoblock cups as compared to 22% in a matched group with modular components. However, González Della Valle et al. (2004) found no difference in wear rates and in prevalence of osteolysis between modular and monoblock acetabular cups (with a 6-year follow-up). The authors concluded that backside wear, which should be present in the modular cups, did not contribute to generation of osteolysis during this intermediate observation time.

There are some potential disadvantages associated with the use of monoblock cups (Sculco 2002). Frequent findings on immediate postoperative radiographs are bone gaps at the apex of the acetabular monoblock cup due to the peripheral press-fit rim (Sculco 2002, Gruen et al. 2005, Macheras et al. 2006). Other possible causes for these findings may be the fact that the liner and the shell are produced as one piece, not allowing visualization of dome contact when seating the cup in the acetabulum.

Our results showed that patients operated with 28-mm femoral heads had a lower risk of revision than patients who underwent surgery with 22-mm heads. There is evidence in the literature that small prosthesis heads increase the risk of hip dislocation (Bystrom et al. 2003). Data from the Australian Joint Replacement Registry showed that there is a statistically significant association between small femoral head diameter and increased risk of revision for dislocation in uncemented cups (Conroy et al. 2008).

High wear rates and a high frequency of retroacetabular osteolysis were recognized as an important reason for premature implant failure in 3 different modular cup designs, with a minimum 12-year follow-up (Hallan et al. 2006). Unsatisfactory results were also reported after the use of 111 modular uncemented ABG-1 cups: here, 12 cups were revised due to retroacetabular osteolysis and almost half of the unrevised cups showed signs of asymptomatic osteolysis at follow-up (Bidar et al. 2009). Inferior long-term survival rates of 9,113 uncemented modular acetabular components were described in a registry-based analysis, where the 10-year survival estimates ranged from 81% to 92% with revision for any reason as the endpoint (Hallan et al. 2010). The authors stated that most acetabular components performed well up to 7 years, but that revision rates increased afterwards. In that study, the modular Trilogy cup showed a 7-year survival rate of 96%, which is comparable to our our medium-term survival data. Valle et al. (2004) followed 271 patients operated with the modular Trilogy cup and found that 98% of them had retention of the cup with good or excellent clinical results at a follow-up of at least 4 years.

Over the last decade, highly crosslinked polyethylene has been introduced to THA surgery with the aim of reducing wear particles. Promising results have been described by several authors. Dorr et al. (2005) found a marked reduction in metal head penetration at 5 years and Bragdon et al. (2007) reported low wear rates at 6 years of follow-up. Our results should be interpreted in the knowledge that highly crosslinked polyethylene was available in more than half of the modular cups but not for monoblock cups.

The main shortcoming of our study was the lack of long-term follow-up data. Revision due to wear of polyethylene, aseptic loosening, and acetabular osteolysis may increase during prolonged follow-up. The number of revisions of monoblock cups was small (n = 8). Still, the lack of statistical significance concerning revision risk does not necessarily imply that the monoblock design has no effect; that is, there may have been type-II error due to the limited number of cases available in the study group and comparatively short follow-up.

An obvious shortcoming of reports from national registries is the uncertainty of adequate reporting from different centers. Also, the failure endpoint currently used (revision) is clear and precise but limited, as it depends on a single surgeon's clinical decision. Furthermore, a potential bias could distort our findings: in the monoblock group, median age was lower and the frequency of non-primary osteoarthritis was higher than in the control group. Both of these factors are known to increase the rate of early loosening, and although we tried to correct for this potential confounder by performing multiple Cox regression analysis, a certain amount of uncertainty remains. The main strength of our study is that it used population-based prospective observational data with excellent compliance. Our data on survival and revisions appear to be rather complete.

Our choice of control group also warrants discussion. We found it reasonable to select a modular uncemented cup that was frequently used in Sweden, and we chose the Trilogy cup without hydroxyapatite coating. This cup has shown an above-average performance in the Swedish Hip Arthroplasty Register, and the comparison of the investigated monoblock designs with this specific cup has probably contributed to the failure to falsify our null-hypothesis of no significant difference between groups. On the other hand, in order to prove the superiority of its concept, the monoblock designs would have to outperform the best modular cups, and this was obviously not the case in our material.

In conclusion, we could not find any clinically relevant difference in revision risk between monoblock and modular acetabular cups in the medium term. Further review of the current patient population is warranted, to determine the long-term durability and risk of revision compared to modular acetabular composites.
